# Lipid-lowering therapy omission at discharge and one-year outcomes after percutaneous coronary intervention: a real-world analysis of overall and elderly patients

**DOI:** 10.1016/j.ijcha.2026.101961

**Published:** 2026-06-24

**Authors:** Denise A.M. Peeters, Eva C.I. Woelders, Sanne Janssen, Jaouad Azzahhafi, Dean R.P.P. Chan Pin Yin, Wout W.A. van den Broek, Qiu Ying F. van de Pol, Aysun Cetinyurek-Yavuz, Patty J.C. Winkler, Peter Damman, Jasper J.P. Luijkx, Wouter S. Remkes, Arnoud W.J. van't Hof, Jurriën M. ten Berg, Robert Jan M. van Geuns

**Affiliations:** a Department of Cardiology, Radboud University Medical Centre, Nijmegen, the Netherlands; b Department of Cardiology, Zuyderland Medical Centre, Heerlen, the Netherlands; c Department of Cardiology, St Antonius Hospital, Nieuwegein, the Netherlands; d IQ Health, Radboud University Medical Centre, Nijmegen, the Netherlands; e Department of Cardiology, Maastricht University Medical Centre, Maastricht, the Netherlands; f Department of Cardiology, VieCuri Medical Centre, Venlo, the Netherlands

**Keywords:** Secondary prevention, Percutaneous coronary intervention, Lipid lowering therapy, MACCE, Elderly

## Abstract

**Background:**

Lipid-lowering therapy (LLT) is recommended in all patients undergoing percutaneous coronary intervention (PCI), but its prescription remains suboptimal. Omission of LLT, particularly in elderly patients, is less well studied. We investigated the prevalence of LLT omission and its association with one-year major adverse cardiovascular and cerebral events (MACCE) in a real-world ACS population, with prespecified focus on elderly through a subgroup analysis.

**Methods:**

Data from the South-east Netherlands Heart Registry (ZON-HR) were merged with the Future Optimal Research and Care Evaluation (FORCE) ACS registry. ACS patients undergoing PCI with known discharge medication and one-year follow-up were included. Associations between LLT prescription at discharge and MACCE, individual components and all-cause death were compared with cox proportional hazard models.

**Results:**

Of 9.495 patients (mean age 65.8, 73.2% male), 720 (7.6%) did not receive LLT. In the elderly population (≥70) this was even higher (10%). Patients without LLT had a higher burden of risk factors. At one year, LLT omission was associated with a higher hazard of MACCE (adjusted HR = 2.70, 95%CI: 2.29–3.18). A comparable association was observed in elderly (adjusted HR of 2.70, 95%CI: 2.18–3.34). Absolute event rates were higher in elderly compared with younger patients (11.4% vs. 7.1%, *p* < 0.001).

**Conclusion:**

In this real-world ACS population, omission of LLT at discharge was common and associated with higher rates of MACCE at one year. While relative associations were similar across age groups, elderly patients experienced higher absolute event rates. These findings may reflect both gaps in care and underlying patient vulnerability.

## Graphical abstract

MACCE = major adverse cardiovascular and cerebrovascular events, HR = hazard ratio. Created with biorender.

**Figure:**
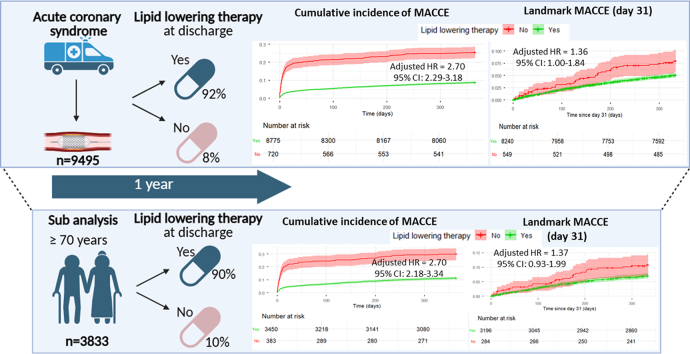

All authors take responsibility for all aspects of the reliability and freedom from bias of the data presented and their discussed interpretation.

## 1 Introduction

Ischemic heart disease remains the leading cause of death worldwide, predominantly driven by atherosclerotic cardiovascular disease [1], [2]. In patients with acute coronary syndrome (ACS), revascularisation with percutaneous coronary intervention (PCI) improves survival and reduces recurrent infarction, whereas in chronic coronary syndrome (CCS) it mainly provides symptom relief [3], [4]. However, even after PCI, the risk of recurrent ischemic coronary events remains higher in patients with ACS than in those with CCS [5].

To prevent recurrent ischemic coronary events, secondary prevention includes high-intensity statins to reduce low-density lipoprotein cholesterol (LDL-C) levels according to European and American guidelines [6], [7], [8]. If statins are not tolerated, other lipid-lowering therapy (LLT), including ezetimibe and proprotein convertase subtilisin/kexin type 9 (PCSK9) inhibitor, should be prescribed. Nevertheless, real-world adherence to LLT recommendations is suboptimal, and a considerable proportion of patients, particularly older patients, are discharged without LLT [9], [10], [11], [12]. One possible explanation is that physicians may be more reluctant to extrapolate evidence from randomized controlled trials (RCTs) to older and potentially frailer patients [13], [14].

The efficacy of LLT in reducing cardiovascular events and mortality has been demonstrated in RCT's [15]. These beneficial effects have also consistently been shown in older patients [16]. However, frail elderly patients encountered in routine ACS care are often underrepresented in randomized trials [17]. In addition, the RCTs assess efficacy in highly standardized and controlled settings, therefore, those results might not reflect the effects of the treatment in daily practice [18].

Real-world data on the association between LLT omission and clinical outcomes after ACS remain limited, particularly in elderly patients. Although the SWEDEHEART registry demonstrated favourable outcomes with LDL-C reduction and LLT, only patients younger than 75 years were included [19].

The aim of this study was to investigate the prevalence of LLT omission and the association between LLT omission and major adverse cardiovascular and cerebrovascular events (MACCE) after PCI in a complete real-world ACS population. Data from two large Dutch, prospective, multicentre registries were used, enabling evaluation of the association between LLT omission on both cardiovascular and all-cause mortality, as well as on other less common individual outcomes such as MI and unplanned or urgent revascularization. Additional subgroup analyses were performed in older patients (>70 years).

## 2 Methods

### 2.1 Study design

To investigate the association between LLT omission at discharge and clinical outcomes after PCI in a real-world ACS population, observational data from the South-East Netherlands Heart Registry (*Zuid-Oost Nederland Hart Registratie*, ZON-HR) was merged with data from the Future Optimal Research and Care Evaluation in patients with Acute Coronary Syndrome (FORCE-ACS) registry. The designs of the registries, including governance and quality control have been described previously [20], [21]. In short, both prospective multicentre registries are ongoing, designed for quality control and improvement of current clinical practice. The ZON-HR includes all patients who underwent PCI since November 2020 in four PCI-centres. The FORCE-ACS includes patients who were admitted with suspected ACS since January 2015 in nine non-interventional and interventional cardiac centres. Patient characteristics, medical history, procedural characteristics, lab values, cardiac medication, and clinical outcomes were extracted from electronic medical records and via patient questionnaires. Both registries were approved by the medical ethics committee and protocols conform to the ethical guidelines of the 1975 Declaration of Helsinki.

### 2.2 Study population

Data from patients with one year follow-up from both registries were harmonised and merged. First, data quality was assessed internally. Next, all variables available in both datasets with comparable definitions and with less than 30% missing values were included. Outcomes were recoded into the same values and afterwards merged into one dataset. Patients included in this analysis underwent PCI for ACS and had known discharge medication. ACS was defined according to the clinical diagnosis recorded in the registries and included ST-elevation MI, Non-ST-elevation MI, and unstable angina pectoris. Out of the 15.526 patients in the combined dataset, 6.031 (38.8%) patients were excluded for the following reasons: (1) no PCI, *n* = 3.443 (22.2%), (2) unknown discharge medication and this also included patients who died during index hospitalization, *n* = 649 (4.2%), (3) no ACS, *n* = 1.939 (12.5%) (supplemental fig. 1). Missing discharge medication information was mainly observed in patients who were transferred to another hospital after PCI and discharged there. To reduce missingness, patients received a questionnaire shortly after PCI that included questions regarding discharge medication. Nevertheless, discharge medication remained unavailable in a subset of patients. The ZON-HR and FORCE-ACS registries include patients from different participating hospital networks in separate regions of the Netherlands. As there was no overlap in participating hospitals between the registries, duplicate patient inclusion would be improbable.

### 2.3 Objectives

1.) Prevalence of patients without LLT at discharge in the overall PCI ACS population and across different age groups.
2.) Association between LLT omission and MACCE, individual MACCE components and all-cause death, one year after PCI in both the complete PCI ACS population and in the older population with patients ≥70 years.

### 2.4 Outcomes

The primary outcome, MACCE, was defined as a composite of cardiovascular mortality, MI, ischaemic stroke, stent thrombosis, and unplanned (ZON-HR) or urgent (FORCE-ACS) revascularization. Cardiovascular death was defined according to the Academic Research Consortium-2 (ARC-2) criteria [22]. MI was defined according to the Fourth Universal Definition of MI [23]. Ischemic stroke was defined as a neurologist-confirmed permanent neurological deficit attributable to focal cerebral, spinal, or retinal ischemia. Unplanned revascularization was defined as a repeat PCI of a specific vessel that had not been scheduled at index procedure, whereas urgent revascularization was defined according to the ARC-2 criteria [22]. The use of these slightly different definitions may have introduced some heterogeneity in the revascularization outcome.

Secondary outcomes consisted of the individual MACCE components and all-cause mortality. Cardiovascular mortality was classified according to ARC-2 criteria using available clinical records. In the absence of documented information regarding the cause of death in the electronic patient file, deaths were classified as cardiovascular. This occurred in approximately 20% of all deaths. All clinical events were verified by the investigators using discharge letters and relevant medical records. In addition, 20% of all events in the ZON-HR were independently adjudicated by a clinical event committee.

### 2.5 Data analysis

Patients were divided into two groups: patients with LLT at discharge and patients without LLT at discharge. LLT was defined as having at least one of the following therapies; statin, ezetimibe, or PCSK9-inhibitor. Categorical variables were expressed as number (percentage) and differences between groups were analyzed with chi-square test. Differences between groups for continuous variables were evaluated with the independent sample *t*-test and expressed as mean ± standard deviation. A 2-tailed *p*-value of <0.05 was considered to be statistically significant. Prescription of LLT in different age groups was analyzed with chi-square test. Linear-by-Linear Association chi-square test was applied to assess whether there was a linear trend. For this analysis, age was categorized starting at ≤50 years with steps of five years per category ending with the category >80 years of age. A sub-analysis was performed for the younger age categories (50–70 years).

Multiple imputation using the Markov Chain Monte Carlo method was performed in SPSS (10 imputed datasets) for baseline variables with less than 30% missing data. Results from imputed datasets were pooled using Rubin's rules. Multivariable adjusted Cox proportional hazard regression was performed to evaluate the association between LLT omission and primary and secondary outcomes, including MACCE, the individual components of MACCE and all-cause death in the complete PCI ACS population and in the elderly subgroup. All baseline variables with a *p-*value <0.3 for between-group differences were included as covariates in the model. The covariates included in each multivariable model are specified in the footnotes of the relevant tables and figures. Log-rank test was used to compare group differences. Absolute 1-year risks of MACCE and MI were estimated using Kaplan-Meier survival analysis.

Sensitivity landmark analyses for cardiovascular death and MACCE from day 31 till one year was performed, which included patients who were still alive after one month, to reduce the influence of early mortality and baseline clinical instability on the observed associations. The landmark analysis was adjusted for baseline variables with a *p-*value <0.3 between the two groups in this landmark cohort and backward variable selection was applied. Additionally, propensity score weighting (inverse probability weighting) was performed in the overall population to balance baseline covariates between patients with and without LLT as an additional quality check to assess consistency with the adjusted models. Weighted Cox regression models were used to estimate pooled hazard ratios for cardiovascular death and MACCE.

Furthermore, a subgroup analysis was conducted in patients aged ≥70 years to explore consistency of associations in older populations. Predictors for receiving no LLT were established with a multivariable binary logistic regression, where variables with a *p* < 0.1 in the univariable regression were included. IBM SPSS Statistics (version 29) was used for statistical analysis and figures were made with R studio (version 2024.04).

## 3 Results

Table 1 shows the baseline characteristics of the included patients (*n* = 9.495). The mean age at index PCI was 65.8 ± 11.7 years and 73.2% were male. No LLT at discharge was observed in 720 patients (7.6%). Patients without LLT at discharge were older (69.3 years vs 65.5 years, *p* < 0.001), more often female (32.5% vs 26.4%, *p* < 0.001), had more often a history of stroke or transient ischemic attack (TIA) (10.6% vs 7.1%, *p* < 0.001), and a higher prevalence of cardiovascular risk factors. They also had a lower estimated glomerular filtration rate (eGFR) compared to patients with LLT (70.3 ± 24.5 vs. 77.8 ± 20.3, *p* < 0.001). No differences in LDL-C at baseline were observed. Patients without LLT at discharge more often underwent left main or multivessel PCI at the index procedure.

**Table 1:** Baseline characteristics.

	Total (*n* = 9.495)	LLT (*n* = 8.775)	No LLT (*n* = 720)	*p-*value
Age	65.8 ± 11.7	65.5 ± 11.6	69.3 ± 12.2	<0.001
Male	6.946 (73.2%)	6.460 (73.6%)	486 (67.5%)	<0.001
BMI	27.6 ± 4.5	27.6 ± 4.5	27.1 ± 4.4	0.005
Medical history				
History of MI	1878 (20.0%)	1724 (19.8%)	154 (21.8%)	0.208
History of PCI	1.938 (20.5%)	1.779 (20.3%)	159 (22.5%)	0.180
History of CABG	615 (6.5%)	557 (6.4%)	58 (8.1%)	0.076
History of stroke/TIA	698 (7.4%)	622 (7.1%)	76 (10.6%)	<0.001
PAD	671 (7.2%)	601 (7.0%)	70 (10.0%)	0.003
Cardiovascular risk factors				
Hypertension	4.818 (52.6%)	4.423 (52.1%)	395 (59.4%)	<0.001
Diabetes	1.740 (18.5%)	1.566 (18.0%)	174 (25.3%)	<0.001
Smoking Current Last year	2.821 (31.6%) 140 (7.7%)	2.656 (31.9%) 135 (8.0%)	165 (26.5%) 5 (4.0%)	0.005 0.111
PCI indication UAP/NSTEMI STEMI	4.698 (49.7%) 4.747 (50.3%)	4.343 (49.7%) 4.394 (50.3%)	355 (50.1%) 353 (49.9%)	0.825
Dialysis	46 (0.5%)	33 (0.4%)	13 (1.8%)	<0.01
Shock	219 (2.4%)	171 (2.0%)	48 (7.1%)	<0.001
OHCA	428 (4.5%)	347 (4.0%)	81 (11.4%)	<0.001
Laboratory blood tests				
LDL-C (mmol/L) eGFR (ml/min/1,73 m2)	3.1 ± 1.1 77.3 ± 20.7	3.1 ± 1.1 77.8 ± 20.3	3.0 ± 1.1 70.3 ± 24.5	0.599 <0.001
PCI characteristics				
Multivessel PCI LM	915 (9.6%) 319 (3.4%)	822 (9.4%) 275 (3.1%)	93 (12.9%) 44 (6.1%)	0.002 <0.001
Discharge medication				
LLT Statins Ezetimibe PCSK9-inhibitor	8.594 (90.5%) 805 (8.5%) 91 (1.0%)	8.594 (97.9%) 805 (9.2%) 91 (1.0%)	0 (0%) 0 (0%) 0 (0%)	<0.001 <0.001 0.006
Antithrombotic therapy Acetylsalicylic acid P2Y12-inhibitor anticoagulant	88.3% 97.3% 14.6%	7929 (90.4%) 8683 (99.0%) 1246 (14.2%)	459 (63.7%) 557 (77.4%) 141 (19.6%)	<0.001 <0.001 <0.001

LLT = lipid lowering therapy; BMI = body mass index; MI = myocardial infarction; PCI = percutaneous coronary intervention; CABG = coronary artery bypass graft; PAD = peripheral arterial disease; TIA = transient ischemic attack; UAP = unstable angina pectoris; NSTEMI = non-ST- segment elevation myocardial infarction; STEMI = ST-segment elevation myocardial infarction; OHCA = out of hospital cardiac arrest; LDL-C = low-density lipoprotein-cholesterol; eGFR = estimated glomerular filtration rate; LM = left main; PCSK9 = proprotein convertase subtilisin kexin type 9.

Regarding the type of LLT at discharge, statins alone or in combination were prescribed in the majority of the patients followed by ezetimibe and PCSK9-inhibitor (90.5%, 8.5%, 1%, respectively). In total, 7.4% of the patients received combination therapy at discharge where most patients (*n* = 623) received ezetimibe in addition to statin therapy (88.8%).

### 3.1 Prescription of lipid lowering therapy in different age groups

**Fig. 1:**
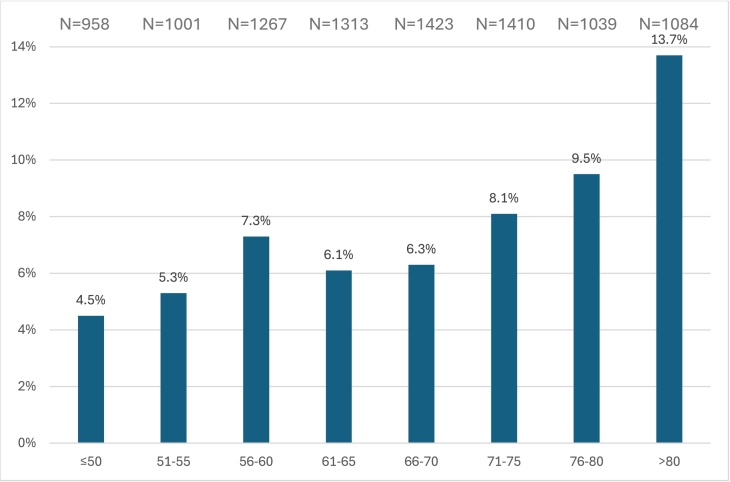
% patients without lipid lowering therapy at discharge across age categories.

Fig. 1 shows the percentage of patients without prescribed LLT at discharge across age categories. The highest percentage was observed in those aged >80 years (13.7%), and the lowest in patients ≤50 years (4.5%, *p* < 0.001). Across all age categories, a significant linear trend was observed between increasing age and lower prescription of LLT at discharge (Linear-by-Linear Association chi-square test, *p* < 0.001). When restricting the analyses to the younger age categories (≤ 70 years), the linear trend was no longer significant (*p* = 0.071).

### 3.2 Clinical outcomes

**Fig. 2:**
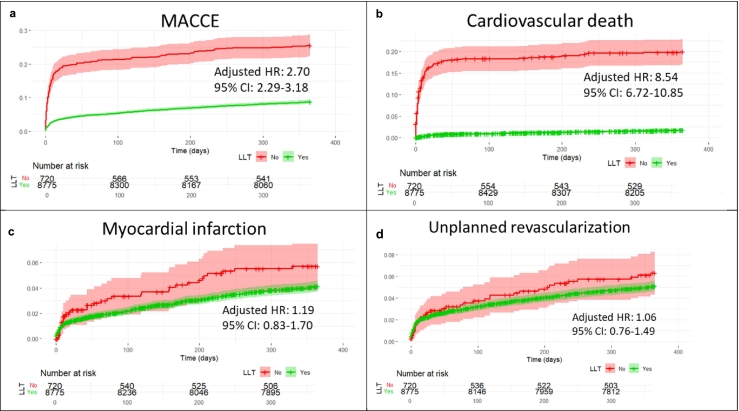
Cumulative incidence of clinical outcomes in patients without LLT compared to patients with LLT one year after PCI. MACCE = major adverse cardiovascular and cerebral events. Adjusted for age, sex, BMI, smoking, diabetes, hypertension, estimated glomerular filtration rate, previous coronary artery bypass graft, previous cerebrovascular accident/transient ischemic attack, peripheral artery disease, percutaneous coronary intervention of left main and multivessel percutaneous coronary intervention.

Patients discharged without LLT were associated with a higher incidence of MACCE within one year after PCI (184/720 [25.6%] vs. 771/8775 [8.8%]). After adjustment for covariates, the association remained significant (adjusted HR = 2.70, 95% CI: 2.29–3.18) (Fig. 2A). The observed association with MACCE was largely attributable to the higher rate of cardiovascular death (adjusted HR = 8.54, 95% CI: 6.72–10.85) (Fig. 2B). For MI, a higher incidence was observed in patients without LLT in the unadjusted model (HR = 1.39, 95% CI: 0.97–1.98), but this difference was not statistically significant after adjustment (adjusted HR = 1.19, 95% CI: 0.83–1.70) (Fig. 2C). The absolute risk for MI at one year was 3.4%. No differences were observed for unplanned or urgent revascularization one year after PCI (adjusted HR = 1.06, 95% CI: 0.76–1.49) (Fig. 2D).

An association between LLT omission and all-cause mortality remained after multivariable adjustment (adjusted HR = 5.55, 95% CI: 4.55–6.77). In the unadjusted model, a 2.17-fold higher risk of ischemic stroke was observed in patients without LLT (95% CI: 1.19–3.99), but no significant difference in the adjusted model was measured (HR = 1.62, 95% CI: 0.88–3.00) (supplemental Table 1).

In the landmark analysis from day 31 onward, 9.284 patients were included out of whom 8.692 received LLT at discharge. Baseline characteristics were comparable to those of the overall study population, except that patients without LLT less frequently presented with STEMI and more often had a history of MI and PCI (supplemental table 2). Both the association with cardiovascular death (HR = 3.20, 95% CI: 1.93–5.32) and MACCE (HR = 1.61, 95% CI: 1.19–2.18) remained statistically significant, but a considerable reduction in HR was observed. After adjustment, a significant difference for cardiovascular death (adjusted HR = 2.07, 95% CI: 1.23–3.50) was observed, whereas the association with MACCE was substantially attenuated (adjusted HR = 1.36, 95% CI: 1.00–1.84). Propensity score-weighted analyses showed a similar direction of association, although the magnitude differed (Supplemental table 3).

### 3.3 Lipid lowering therapy in elderly patients

The ACS PCI cohort in this study consisted of 3.833 patients (40.4%) aged 70 years or older. The mean age of this older cohort was 77.3 ± 5.3 years and 65.8% was male. A quarter of these patients had a previous PCI and the mean LDL-C value was 2.8 ± 1.1 mmol/L. In this elderly subgroup, 10% (*n* = 383) of the patients did not receive LLT. Compared with those who did, patients without LLT were slightly older (78.8 ± 5.9 vs 77.1 ± 5.3, *p* < 0.001), had a higher prevalence of stroke or TIA (15.4% vs 11.6%, *p* = 0.032), diabetes (27.0% vs 22.0%, *p* = 0.027), higher LDL-C (3.0 ± 1.1 vs 2.8 ± 1.1, *p* = 0.005) and lower eGFR levels (61.3 ± 22.7 vs 68.6 ± 20.4, *p* < 0.001) (Supplemental Table 4). Older age, higher LDL-C baseline values, and lower eGFR values were independent predictors for receiving no LLT at discharge in the older population (Table 2).

**Table 2:** Multivariable binary logistic regression of predictors for receiving no lipid lowering therapy at discharge in patients ≥70 years.

Variable	OR	95% CI
Age	1.04	1.02–1.06
Male	1.04	0.83–1.30
History of stroke/TIA	1.26	0.92–1.72
PAD	1.20	0.87–1.64
Diabetes	1.21	0.94–1.56
LDL-C	1.18	1.05–1.33
eGFR	0.99	0.98–0.99

TIA = transient ischemic attack; PAD = peripheral arterial disease; LDL-C = low density lipoprotein- Cholesterol; eGFR = estimated glomerular filtration rate.

### 3.4 Clinical outcomes in patients without LLT aged 70 or older

Older patients discharged without LLT exhibited a higher incidence of MACCE at one year compared with those discharged with LLT (115/383 [30.0%] vs. 394/3450 [11.4%]). After adjustment, the association remained significant (adjusted HR = 2.70, 95% CI: 2.18–3.34). Higher adjusted hazards of cardiovascular death (adjusted HR = 6.60, 95% CI: 4.92–8.85) and all-cause mortality (adjusted HR = 4.33, 95% CI: 3.39–5.52) were observed among older patients discharged without LLT. Higher risk for the individual outcome MI and ischemic stroke were observed in the unadjusted model (HR = 1.62, 95% CI: 1.07–2.47 and HR = 2.09, 95% CI: 1.06–4.11, respectively). The adjusted HR for MI showed a statistically non-significant difference (adjusted HR = 1.53, 95% CI: 1.00–2.34) (Table 3). The absolute 1-year MACCE rate was 11.4% and the corresponding rate in patients <70 years was 7.1%, *p* < 0.001. The absolute risk for MI at one year was 4.4% in the elderly and 2.1% in the younger patients, *p* < 0.001.

**Table 3:** No LLT at discharge in patients ≥70 year and clinical outcome.

	No LLT (n = 383)	LLT (*n* = 3450)	HR (95%CI)	*p*-value
MACCE Unadjusted Multivariable	115/383 (30.0%)	394/3450 (11.4%)	3.07 (2.49–3.78) 2.70 (2.18–3.34)	<0.001 <0.001
All-cause death Unadjusted Multivariable	104/383 (27.2%)	207/3450 (6.0%)	5.44 (4.30–6.89) 4.33 (3.39–5.52)	<0.001 <0.001
Cardiovascular death Unadjusted Multivariable	87/383 (22.7%)	107/3450 (3.1%)	8.59 (6.47–11.40) 6.60 (4.92–8.85)	<0.001 <0.001
Myocardial infarction Unadjusted Multivariable	25/383 (6.5%)	169/3450 (4.9%)	1.62 (1.07–2.47) 1.53 (1.00–2.34)	0.024 0.052
Stent thrombosis Unadjusted Multivariable	6/383 (1.6%)	29/3450 (0.8%)	2.14 (0.89–5.17) 2.00 (0.82–4.91)	0.089 0.13
Ischaemic stroke Unadjusted Multivariable	10/383 (2.6%)	52/3450 (1.5%)	2.09 (1.06–4.11) 1.78 (0.90–3.54)	0.033 0.098
Unplanned/ urgent revascularization Unadjusted Multivariable	24/383 (6.3%)	181/3450 (5.2%)	1.42 (0.93–2.17) 1.34 (0.87–2.06)	0.107 0.185

Associations between no LLT at discharge and clinical outcomes. Adjusted for age, sex, diabetes, estimated glomerular filtration rate, low density lipoprotein cholesterol, peripheral artery disease, previous cerebrovascular accident/ transient ischemic attack, dialysis, out of hospital cardiac arrest and shock.

In the landmark analysis from day 31 onward, 3.696 patients were included out of whom 3.391 received LLT at discharge. Baseline characteristics were comparable to those of the overall older study population, except for the percentage of patients who had an out of hospital cardiac arrest which became lower in the landmark cohort in the group without LLT at discharge (*n* = 24 (6.3%) vs *n* = 9 (3.0%) (supplemental table 5). Both the association with cardiovascular death (HR = 2.40, 95% CI: 1.32–4.37) and MACCE (HR = 1.57, 95% CI: 1.08–2.27) remained significant, however the magnitude of the association was substantially attenuated. After adjustment for covariates, no significant difference were observed for cardiovascular death (adjusted HR = 1.75, 95% CI: 0.94–3.23) and MACCE (adjusted HR = 1.37, 95% CI: 0.93–1.99) (graphical abstract).

## 4 Discussion

The current study evaluated the association between LLT omission at discharge and clinical outcomes in nearly 10.000 ACS patients undergoing PCI. Two large Dutch, multicentre, prospective registries were used. The follow-up was one year. The main findings were: 1) 7.6% of the ACS patients did not receive LLT at discharge, increasing to 10% in patients aged ≥70 years; 2) LLT omission was associated with a higher incidence of MACCE and cardiovascular death at one year; and 3) While relative associations were comparable across age groups, elderly patients experienced substantially higher absolute event rates, highlighting a potential care gap in a particularly high-risk population.

### 4.1 Comparison with previous studies

In the complete ACS PCI population, the highest incidence among patients without LLT at discharge was observed for MACCE. However, this was mainly driven by the highest relative risk of the individual outcome cardiovascular death. A similar association between more intensive LLT and lower cardiovascular mortality has been reported in the SWEDEHEART registry [19]. However, the steep increase in cardiovascular mortality early after PCI in patients without LLT in our study supports the notion that early risk differences may reflect underlying patient characteristics and clinical instability. Patients with more complex coronary disease, such as multivessel or left main PCI, were less likely to be discharged on LLT. Although data on periprocedural complications were not available, these patients may have experienced greater clinical instability or complications during or shortly after PCI, potentially leading clinicians to defer LLT initiation. Several factors may explain the omission of LLT at discharge. Patients without LLT more frequently presented with severe clinical conditions, including cardiogenic shock, out-of-hospital cardiac arrest, and advanced renal dysfunction, which may have contributed to a shift in treatment focus toward stabilization of acute illness rather than optimization of secondary prevention therapies. Furthermore, physician-level factors, including variability in prescribing practices or concerns regarding tolerability in frail or clinically unstable patients, may have contributed to under-prescription of LLT despite a higher ischemic risk profile.

Importantly, the association between LLT omission and cardiovascular mortality was markedly attenuated in the landmark analysis from day 31 onward, although it remained significant. This suggests that early mortality and immediate clinical instability contribute substantially to the observed association but do not fully explain it. In the elderly subgroup, only the unadjusted association with cardiovascular death remained statistically significant in the landmark analysis. However, the direction of the effect remained consistent across outcomes. The lack of statistical significance after adjustment may reflect reduced power resulting from the smaller number of patients and events. These findings are directionally consistent with evidence from randomized trials demonstrating benefit of statin therapy in older patients [24]. On the other hand, the weakened and non-significant association for MACCE in the elderly population in the landmark analysis suggests that early mortality and acute illness severity may account for a substantial proportion of the observed effect.

Elderly patients are more likely to die from non-cardiovascular causes, which may influence the estimated association with cardiovascular mortality [12], [25]. However, competing mortality alone is unlikely to account for the observed findings.

### 4.2 Individual outcomes

Patients without LLT at discharge had an approximately twofold higher incidence of ischemic stroke compared to patients with LLT. No significant differences were observed for the other individual outcomes. The incidence of MI was numerically higher in patients without LLT, but the difference did not reach statistical significance, while rates of urgent or unplanned revascularization were similar between groups. This might be explained by the different pathophysiological mechanisms and the limited follow-up period of one year in our study. The anti-inflammatory effects may contribute to early plaque stabilization and prevents MIs [26]. The MIRACL trial demonstrated a reduction in recurrent ischemic events after 16 weeks in ACS patients with statin therapy compared to ACS patients without statin therapy [27]. In contrast, reductions in atherosclerotic disease progression may primarily translate into lower revascularization rates over longer follow-up periods. The PROVE-IT-TIMI 22 trial showed a reduction of revascularization in ACS PCI patients on high intensity statins compared to ACS PCI patients on moderate statins after two years [28]. Although the present study focused on LLT omission versus prescription of any LLT, the clinical effect of LLT may vary according to the treatment intensity and regimen, including the addition of ezetimibe or PCSK9-inhibitor. Moreover, the number of patients treated with ezetimibe and/or PCSK9-inhibitors was limited.

### 4.3 Elderly patients

Older patients remain undertreated regarding LLT. In this elderly subgroup, 10% of patients were receiving no LLT while previous RCT's have shown the benefits of LLT for secondary prevention [16]. To date, real-world data on the effects of LLT on clinical outcomes in elderly patients remains limited. The French registry of Acute ST-Elevation and non-ST-elevation Myocardial Infarction (FAST-MI) compared LLT in men and women after MI. There was a mean age difference of almost 7 years between included men and women, where women were older (no high intensity LLT: 67.5 vs 74.4 years; high intensity LLT: 61.2 vs 68.9 years). An adjusted HR of 0.74 was observed for mortality after five years in women with high-dose LLT at discharge compared to women with no high-dose LLT at discharge [29]. We showed that patients ≥70 years old without LLT had a higher adjusted HR for MACCE, all-cause death and cardiovascular death and a higher unadjusted HR for MI and ischemic stroke one year after PCI.

In this elderly subgroup, we additionally observed that patients with higher LDL-C levels were less likely to be discharged on LLT, a finding that appears counterintuitive in the context of secondary prevention. A plausible explanation relates to prior LLT use, which could not be reliably determined in this study. It is likely that a substantial proportion of older patients had already been receiving long-term LLT before PCI, resulting in lower measured LDL-C levels at presentation. Conversely, higher LDL-C levels in elderly patients may reflect the absence of prior LLT exposure rather than a lower need for therapy, thereby creating an apparent inverse association between LDL-C levels and LLT prescription at discharge. In addition, age-related factors such as frailty, multimorbidity, polypharmacy, or concerns regarding drug tolerability may have influenced prescribing decisions in older patients, potentially leading to more conservative LLT use despite elevated LDL-C levels. As pre-admission medication data were unavailable, these interpretations remain speculative. Nevertheless, the observed association between absence of LLT and adverse clinical outcomes in patients aged ≥70 years supports further attention to guideline-directed LLT in this high-risk population.

### 4.4 Limitations

This study has some limitations. As this study was conducted in a predominantly Dutch population, the generalizability of our findings to other racial and ethnic groups may be limited. Residual confounding by indication cannot be excluded, as patients not receiving LLT may systematically differ from those treated, particularly with regard to frailty, clinical instability, or therapeutic goals. Although adjustments and propensity score weighting were applied, unmeasured confounders remain possible. Also, frailty indicators, pre-admission medication, and medication doses were not available, limiting interpretation of treatment decisions. Since data on therapy adherence were unavailable, analyses were performed according to the discharge prescription and should therefore be interpreted as an ‘intention to treat’ approach. Changes in LLT during follow-up, including treatment discontinuation, dose modification, or treatment initiation after discharge, were not captured. Consequently, discharge medication may not accurately reflect long-term LLT exposure, which could have attenuated or otherwise influenced the observed associations. Therefore, our findings should be interpreted primarily as identifying a high-risk and potentially undertreated population rather than demonstrating a direct causal effect of LLT omission on mortality. The relatively modest association with MI compared with the strong association with cardiovascular mortality further supports this interpretation. As detailed cause-specific cardiovascular mortality data were unavailable, it remains unclear whether excess cardiovascular deaths were related to the recurrent ischemic events, heart failure, arrhythmias, procedural complications, or other manifestations of clinical vulnerability.

## 5 Conclusion

Our study in two large Dutch registries including an all comers ACS population undergoing PCI showed that patients who did not receive guideline-directed LLT at discharge experienced higher rates of MACCE and mortality during follow-up. This observation is consistent in patients ≥70 years of age. As the incidence of adverse cardiovascular outcomes increases with age, these findings identify a subgroup of ACS patients, particularly older patients, in whom guideline directed LLT was less frequently prescribed and who experienced substantially higher event rates during follow-up.

## CRediT authorship contribution statement

**Denise A.M. Peeters:** Writing – original draft, Visualization, Methodology, Investigation, Formal analysis, Data curation, Conceptualization. **Eva C.I. Woelders:** Writing – review & editing, Investigation. **Sanne Janssen:** Writing – review & editing, Investigation. **Jaouad Azzahhafi:** Writing – review & editing, Investigation. **Dean R.P.P. Chan Pin Yin:** Writing – review & editing, Investigation. **Wout W.A. van den Broek:** Writing – review & editing, Investigation. **Qiu Ying F. van de Pol:** Writing – review & editing, Investigation. **Aysun Cetinyurek-Yavuz:** Writing – review & editing, Formal analysis. **Patty J.C. Winkler:** Writing – review & editing, Investigation. **Peter Damman:** Writing – review & editing, Supervision, Investigation, Funding acquisition, Conceptualization. **Jasper J.P. Luijkx:** Writing – review & editing, Investigation. **Wouter S. Remkes:** Writing – review & editing, Investigation. **Arnoud W.J. van't Hof:** Writing – review & editing, Supervision, Funding acquisition, Conceptualization. **Jurriën M. ten Berg:** Writing – review & editing, Supervision, Resources, Funding acquisition. **Robert Jan M. van Geuns:** Writing – review & editing, Supervision, Resources, Investigation, Funding acquisition, Conceptualization.

## Funding

The FORCE-ACS registry is supported by grants from ZonMw, the St. Antonius Research Fund, and AstraZeneca. The ZON-HR is supported by unrestricted research grants from AstraZeneca, Sanofi and Boehringer Ingelheim**.**

## Declaration of Competing Interest

The authors declare the following financial interests/personal relationships which may be considered as potential competing interests: Jurrien M. ten Berg reports financial support was provided by Netherlands Organisation for Health Research and Development. Jurrien M. ten Berg reports financial support was provided by St. antonius research fund. Jurrien M. ten Berg reports financial support was provided by AstraZeneca. Robert-Jan M. van Geuns reports financial support was provided by AstraZeneca. Robert-Jan M. van Geuns reports financial support was provided by Sanofi. Arnoud W.J. van ’t Hof reports financial support was provided by Boehringer Ingelheim GmbH. Robert-Jan M. van Geuns reports a relationship with Amgen Inc that includes: funding grants. Robert-Jan M. van Geuns reports a relationship with Infraredx Inc that includes: funding grants and speaking and lecture fees. Robert-Jan M. van Geuns reports a relationship with AstraZeneca that includes: speaking and lecture fees. Robert-Jan M. van Geuns reports a relationship with Abbott Vascular Inc that includes: speaking and lecture fees. Peter Damman reports a relationship with Abbott that includes: funding grants and speaking and lecture fees. Peter Damman reports a relationship with Philips that includes: funding grants and speaking and lecture fees. Peter Damman reports a relationship with Pie Medical Imaging BV that includes: funding grants. Arnoud W.J. van ’t Hof reports a relationship with Abbott that includes: funding grants. Arnoud W.J. van ’t Hof reports a relationship with Medtronic that includes: funding grants. Arnoud W.J. van ’t Hof reports a relationship with AstraZeneca that includes: funding grants. Arnoud W.J. van ’t Hof reports a relationship with CeleCor Therapeutics Inc that includes: consulting or advisory. Patty J.C. Winkler reports a relationship with Abbott that includes: speaking and lecture fees. Jurrien M. ten Berg reports a relationship with Daiichi Sankyo Inc that includes: funding grants and speaking and lecture fees. Jurrien M. ten Berg reports a relationship with AstraZeneca that includes: funding grants and speaking and lecture fees. Jurrien M. ten Berg reports a relationship with CeleCor Therapeutics Inc that includes: speaking and lecture fees. Jurrien M. ten Berg reports a relationship with Boehringer Ingelheim GmbH that includes: speaking and lecture fees. If there are other authors, they declare that they have no known competing financial interests or personal relationships that could have appeared to influence the work reported in this paper.

## Data Availability

The data underlying this article cannot be shared due to reasons of sensitivity and lack of participant consent for public data sharing.

## Appendix A Supplementary data

**Supplementary material 1:** Additional analyses and supporting data, including the study flowchart, clinical outcomes in the overall population, baseline characteristics of patients included in the landmark analyses, propensity score-weighted analyses, and detailed baseline characteristics of the elderly population.
