# Supplementary material for: Lipid-lowering therapy omission at discharge and one-year outcomes after percutaneous coronary intervention: a real-world analysis of overall and elderly patients
Source: Int J Cardiol Heart Vasc. 2026 Jun 24;65:101961. doi: 10.1016/j.ijcha.2026.101961 (PMC13320445; doi:10.1016/j.ijcha.2026.101961)
Supplement: Supplementary file 1 — Supplementary material 1: Additional analyses and supporting data, including the study flowchart, clinical outcomes in the overall population, baseline characteristics of patients included in the landmark analyses, propensity score-weighted analyses, and detailed baseline characteristics of the elderly population. [file mmc1.docx]

# Supplements


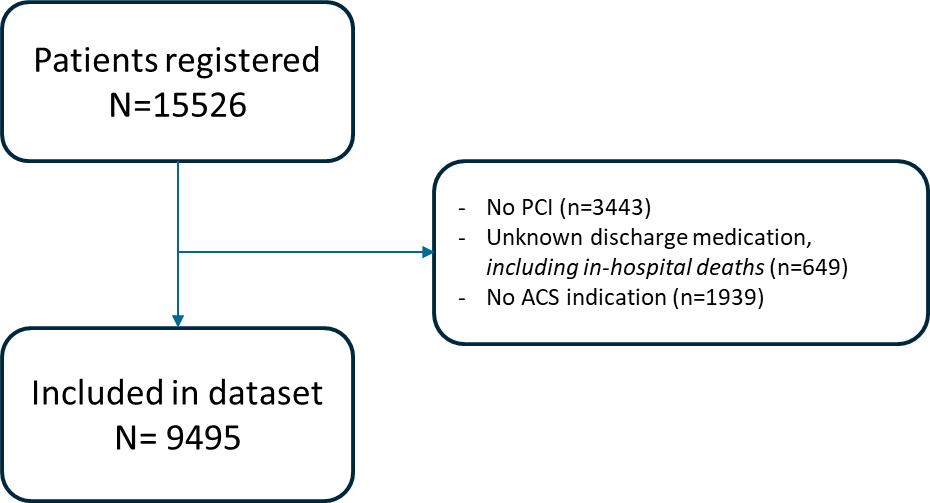


**Supplemental figure 1. Study flowchart**

**Supplemental table 1. No LLT at discharge and clinical outcomes**

|  | **No LLT (n=720)** | **LLT (n=8775)** | **HR (95% CI)** | ***p*-value** |
| --- | --- | --- | --- | --- |
| **MACCE**  Unadjusted  Multivariable | 184/720 (25.6%) | 771/8775 (8.8%) | 3.34 (2.84-3.92)  2.70 (2.29-3.18) | <0.001  <0.001 |
| **All-cause death**  Unadjusted  Multivariable | 163/720 (22.6%) | 282/8775 (3.2%) | 8.38 (6.91-10.16)  5.55 (4.55-6.77) | <0.001  <0.001 |
| **Cardiovascular death**  Unadjusted  Multivariable | 140/720 (19.4%) | 150/8775 (1.7%) | 13.22 (10.50-16.65)  8.54 (6.72-10.85) | <0.001  <0.001 |
| **Myocardial infarction**  Unadjusted  Multivariable | 33/720 (4.6%) | 348/8775 (4.0%) | 1.39 (0.97-1.98)  1.19 (0.83-1.70) | 0.073  0.354 |
| **Stent thrombosis**  Unadjusted  Multivariable | 8/720 (1.1%) | 82/8775 (0.9%) | 1.34 (0.65-2.76)  1.08 (0.52-2.27) | 0.435  0.832 |
| **Ischaemic stroke**  Unadjusted  Multivariable | 12/720 (1.7%) | 81/8775 (0.9%) | 2.17 (1.19-3.99)  1.62 (0.88-3.00) | 0.012  0.121 |
| **Unplanned/ urgent revascularization**  Unadjusted  Multivariable | 37/720 (5.1%) | 437/8775 (5.0%) | 1.21 (0.87-1.70)  1.06 (0.76-1.49) | 0.259  0.735 |
| Associations between no LLT at discharge and clinical outcomes. Adjusted for age, sex, BMI , smoking, diabetes, hypertension, estimated glomerular filtration rate, previous coronary artery bypass graft, previous cerebrovascular accident/transient ischemic attack, peripheral artery disease, percutaneous coronary intervention of left main and multivessel percutaneous coronary intervention, dialysis, out of hospital cardiac arrest and shock. | | | | |

**Supplemental table 2. Baseline differences of patients included in the landmark analysis**

|  | **LLT (n=8.692)** | **No LLT (n=592)** | ***p-*value** |
| --- | --- | --- | --- |
| Age | 65.5 ± 11.6 | 68.9 ± 12.4 | <0.001 |
| Male | 6.400 (73.6%) | 404 (68.2%) | 0.004 |
| BMI | 27.6 ± 4.5 | 27.1 ± 4.4 | 0.009 |
| Medical history | | | |
| History of MI | 1695 (19.7%) | 134 (23.1%) | 0.049 |
| History of PCI | 1.753 (20.2%) | 141 (24.2%) | 0.021 |
| History of CABG | 542 (6.2%) | 48 (8.1%) | 0.073 |
| History of stroke/TIA | 609 (7.0%) | 57 (9.7%) | 0.015 |
| PAD | 585 (6.9%) | 54 (9.4%) | 0.023 |
| Cardiovascular risk factors | | | |
| Hypertension | 4.376 (52.0%) | 327 (59.1%) | <0.001 |
| Diabetes | 1.542 (17.9%) | 139 (24.3%) | <0.001 |
| Smoking  Current  Last year | 2640 (1.5%)  133 (7.9%) | 134 (25.6%)  5 (4.7%) | 0.002  0.221 |
| PCI indication  UAP/NSTEMI  STEMI | 4.301 (49.7%)  4.353 (50.3%) | 328 (56.0%)  258 (44.0%) | 0.003 |
| Dialysis | 32 (0.4%) | 10 (1.7%) | <0.001 |
| Shock | 163 (1.9%) | 20 (3.6%) | 0.007 |
| OHCA | 344 (4.0%) | 37 (6.3%) | 0.005 |
| Laboratory blood tests | | | |
| LDL-C (mmol/L)  eGFR (ml/min/1,73m2) | 3.1 ± 1.1  77.9 ± 20.2 | 3.1 ± 1.1  73.1 ± 24.1 | 0.480  <0.001 |
| PCI characteristics | | | |
| Multivessel PCI  LM | 812 (9.3%)  267 (3.1%) | 74 (12.5%)  27 (4.6%) | 0.011  0.045 |
| LLT= lipid lowering therapy; BMI= body mass index; MI= myocardial infarction; PCI= percutaneous coronary intervention; CABG= coronary artery bypass graft; PAD= peripheral arterial disease; TIA= transient ischemic attack; UAP= unstable angina pectoris; NSTEMI=non-ST- segment elevation myocardial infarction; STEMI= ST-segment elevation myocardial infarction; OHCA= out of hospital cardiac arrest; LDL= low-density lipoprotein Cholesterol; eGFR= estimated glomerular filtration rate; LM= left main. | | | |

**Supplemental table 3. Propensity score-weighted analyses for the overall population**

| **Endpoint** | **Model** | **HR (95% CI)** | ***p*-value** |
| --- | --- | --- | --- |
| **Cardiovascular death** | Propensity score-weighted Cox (ATC) | 7.45 (5.77-9.62) | <0.001 |
| **MACCE** | Propensity score-weighted Cox (ATC) | 2.66 (2.22-3.16) | <0.001 |
| HR= hazard ratio; CI= confidence interval; MACCE= major adverse cardiovascular and cerebrovascular events; ATC= Average Treatment effect in the Controls. | | | |

**Supplemental table 4. Baseline characteristics in patients ≥70 year**

|  | **Total (n=3833)** | **LLT (n=3450)** | **No LLT (n=383)** | ***p-*value** |
| --- | --- | --- | --- | --- |
| Age | 77.3 ± 5.3 | 77.1 ± 5.3 | 78.8± 5.9 | <0.001 |
| Male | 65.8% | 66.3% | 61.9% | 0.084 |
| BMI | 26.8 ± 4.3 | 26.8 ± 4.3 | 26.7 ± 4.2 | 0.805 |
| Medical history | | | |  |
| History of MI | 25.5% | 25.4% | 25.7% | 0.905 |
| History of PCI | 26.2% | 26.3% | 26.1% | 0.956 |
| History of CABG | 10.8% | 10.7% | 11.5% | 0.659 |
| History of stroke/TIA | 12.0% | 11.6% | 15.4% | 0.032 |
| PAD | 11.7% | 11.4% | 14.5% | 0.073 |
| Cardiovascular risk factors | | | |  |
| Hypertension | 64.5% | 64.6% | 64.2% | 0.884 |
| Diabetes | 22.5% | 22.0% | 27.0% | 0.027 |
| Smoking  Current  Last year | 14.4%  3.6% | 14.7%  3.8% | 11.9%  1.3% | 0.161  0.276 |
| PCI indication  UAP/NSTEMI  STEMI | 57.8%  42.2% | 58.1%  41.9% | 55.5%  44.5% | 0.335 |
| Dialysis | 33 (0.9%) | 25 (0.7%) | 8 (2.1%) | 0.006 |
| Shock | 89 (2.3%) | 68 (2.0%) | 21 (5.5%) | <0.001 |
| OHCA | 128 (3.3%) | 104 (3.0%) | 24 (6.3%) | <0.001 |
| Laboratory blood tests | | | |  |
| LDL-C (mmol/L)  eGFR (ml/min/1,73m2) | 2.8 ± 1.1  67.9 ± 20.8 | 2.8 ± 1.1  68.6 ± 20.4 | 3.0 ± 1.1  61.3 ± 22.7 | 0.005  <0.001 |
| PCI characteristics | | | |  |
| Multivessel PCI | 11.6% | 11.3% | 14.1% | 0.105 |
| LM | 5.1% | 4.9% | 6.8% | 0.117 |
| LLT at discharge |  |  |  |  |
| Statins  Ezetimibe  PCSK9-inhibitor | 87.7%  8.5%  0.8% | 97.4%  9.4%  0.9% | 0%  0%  0% | <0.001  <0.001  0.067 |
| LLT= lipid lowering therapy; BMI= body mass index; MI= myocardial infarction; PCI= percutaneous coronary intervention; CABG= coronary artery bypass graft; PAD= peripheral arterial disease; TIA= transient ischemic attack; UAP= unstable angina pectoris; NSTEMI=non-ST- segment elevation myocardial infarction; STEMI= ST-segment elevation myocardial infarction; OHCA= out of hospital cardiac arrest; LDL= low-density lipoprotein Cholesterol; eGFR= estimated glomerular filtration rate; LM= left main; PCSK9= proprotein convertase subtilisin kexin type 9. | | | | |

**Supplemental table 5. Baseline characters of the older patients included in landmark analysis**

|  | **LLT (n=3391)** | **No LLT (n=305)** | ***p*-value** |
| --- | --- | --- | --- |
| Age | 77.1 ± 5.3 | 78.1 ± 5.9 | <0.001 |
| Male | 66.2% | 61.6% | 0.107 |
| BMI | 26.8 ± 4.3 | 26.7 ± 4.1 | 0.805 |
| Medical history | | | |
| History of MI | 25.3% | 27.6% | 0.383 |
| History of PCI | 26.2% | 27.8% | 0.541 |
| History of CABG | 10.6% | 11.5% | 0.651 |
| History of stroke/TIA | 11.5% | 15.3% | 0.047 |
| Cardiovascular risk factors | | | |
| PAD | 11.2% | 13.9% | 0.156 |
| Hypertension | 64.5% | 63.2% | 0.642 |
| Diabetes | 21.7% | 25.0% | 0.193 |
| Smoking  Current  Last year | 14.7%  3.7% | 11.0%  1.6% | 0.098  0.404 |
| PCI indication  UAP/NSTEMI  STEMI | 58.2%  41.8% | 63.8%  36.2% | 0.059 |
| Dialysis | 25 (0.7%) | 6 (2.0%) | 0.024 |
| Shock | 60 (1.8%) | 9 (3.0%) | 0.144 |
| OHCA | 101 (3.0%) | 9 (3.0%) | 0.978 |
| Laboratory blood tests | | | |
| LDL-C (mmol/L)  eGFR (ml/min/1,73m2) | 2.8 ± 1.1  68.6 ± 20.3 | 3.0 ± 1.1  63.3 ± 22.0 | 0.005  <0.001 |
| PCI characteristics | | | |
| Multivessel PCI | 11.2% | 14.4% | 0.091 |
| LM | 4.8% | 6.6% | 0.117 |
| LLT= lipid lowering therapy; BMI= body mass index; MI= myocardial infarction; PCI= percutaneous coronary intervention; CABG= coronary artery bypass graft; PAD= peripheral arterial disease; TIA= transient ischemic attack; UAP= unstable angina pectoris; NSTEMI=non-ST- segment elevation myocardial infarction; STEMI= ST-segment elevation myocardial infarction; OHCA= out of hospital cardiac arrest; LDL= low-density lipoprotein Cholesterol; eGFR= estimated glomerular filtration rate; LM= left main. | | | |
